# Polynomial Phase Estimation Based on Adaptive Short-Time Fourier Transform

**DOI:** 10.3390/s18020568

**Published:** 2018-02-13

**Authors:** Fulong Jing, Chunjie Zhang, Weijian Si, Yu Wang, Shuhong Jiao

**Affiliations:** College of Information and Communication Engineering, Harbin Engineering University, Harbin 150001, China; jing_fl@hrbeu.edu.cn (F.J.); swj0418@263.net (W.S.); wangyu1256@hrbeu.edu.cn (Y.W.); jiaoshuhong@hrbeu.edu.cn (S.J.)

**Keywords:** adaptive short-time Fourier transform, instantaneous frequency gradient estimation, polynomial phase signals, parameters estimation, time–frequency signal analysis

## Abstract

Polynomial phase signals (PPSs) have numerous applications in many fields including radar, sonar, geophysics, and radio communication systems. Therefore, estimation of PPS coefficients is very important. In this paper, a novel approach for PPS parameters estimation based on adaptive short-time Fourier transform (ASTFT), called the PPS-ASTFT estimator, is proposed. Using the PPS-ASTFT estimator, both one-dimensional and multi-dimensional searches and error propagation problems, which widely exist in PPSs field, are avoided. In the proposed algorithm, the instantaneous frequency (IF) is estimated by S-transform (ST), which can preserve information on signal phase and provide a variable resolution similar to the wavelet transform (WT). The width of the ASTFT analysis window is equal to the local stationary length, which is measured by the instantaneous frequency gradient (IFG). The IFG is calculated by the principal component analysis (PCA), which is robust to the noise. Moreover, to improve estimation accuracy, a refinement strategy is presented to estimate signal parameters. Since the PPS-ASTFT avoids parameter search, the proposed algorithm can be computed in a reasonable amount of time. The estimation performance, computational cost, and implementation of the PPS-ASTFT are also analyzed. The conducted numerical simulations support our theoretical results and demonstrate an excellent statistical performance of the proposed algorithm.

## 1. Introduction

In the past few decades, the non-stationary signal processing has been a very important research field [1,2,3,4], especially estimating polynomial phase signals (PPSs). The PPSs arise in many fields including radar, sonar, radio communication, biology, and geophysics systems [5,6,7]. The *M*th order PPS can be described by the following model:

(1) $$x\left(t\right)=A\exp\left(j\sum_{k=1}^{M}a_{k}t^{k}/k+ja_{0}\right),\;t\in \left[-T/2,T/2\right),$$

where *A* and $a_{m}$ denote the amplitude and phase parameters, respectively, *T* is the signal duration, and *j* is the imaginary unit. Recently, numerous methods for PPS parameters estimation have been developed. The most popular methods can be roughly grouped into three classes: estimators based on phase unwrapping (PU), estimators based on phase-differentiation (PD), and estimators based on time-frequency representation (TFR).

The PU-based estimators mainly include the Kitchen’s unwrapping estimator [8], the SEparate-EStimate (SEES) estimator [9], and the least square unwrapping (LSU) estimator [10]. The main drawback of the PU-based methods is that they are sensitive to the noise influence, while recent development in this field has improved accuracy but this has been paid by high calculation demands.

Compared to the PU-based estimators, the PD-based estimators are simpler and can be easily implemented. In the PD-based estimator, the PPS order is decreased by the PD operation until a complex sinusoid signal is obtained. The frequency of the sinusoid signal is proportional to the highest order PPS coefficient, and the original signal is dechirped by the highest order PPS coefficient. The other parameters are estimated by repeating the same procedure. The PD-based estimators mainly include the high-order ambiguity function (HAF) [11,12], the product HAF (PHAF) [13], the integrated generalized ambiguity function (IGAF) [14], the cubic phase function (CPF) [15,16], the hybrid CPF-HAF [17], and the product CPF-HAF (PCPF-HAF) [17]. Among these methods, the PHAF and the PCPF-HAF are the most representative ones, since these two methods show much better identifiability and noise rejection capability than the other methods. Although the PD-based methods are widely used and easily implemented, they still suffer from numerous problems. Firstly, the signal-to-noise ratio (SNR) threshold and mean-squared error (MSE) are increased because every PD operation increases the number of noise-influenced terms and reduces the signal length. Secondly, for multicomponent PPSs, the identifiability problem still exists in most PD-based estimators. Finally, the PD-based estimators suffer from error propagation.

The representative estimator of TFR-based estimators is the quasi-maximum likelihood (QML) estimator [18,19,20], which powerfully avoids the disadvantages of PD-based estimators and PU-based estimators. The main advantages of the QML estimators are as follows. First, the coarse estimation, which is based on the short-time Frequency transform (STFT) [6,21] and the polynomial regression [18,22], is robust to noise influence. Second, by applying the phase unwrapping, filtering and fitting to the dechirped PPS signal, a high SNR threshold can be achieved. Finally, the optimal PPS coefficients are estimated by one-dimensional search over the STFT window widths, which is similar to the maximum likelihood (ML). Thus, the QML can achieve the Cramer–Rao lower bound (CRLB) [23,24] for low SNR values even for high-order PPS. Furthermore, because all parameters are estimated simultaneously, there is no error propagation problem. Although the QML can achieve excellent performance, searching over the window widths still induce high computational cost.

In accordance with the above analysis, a novel TFR-based estimator based on the adaptive short-time Fourier transform (ASTFT) is developed. First, the instantaneous frequency (IF) is coarsely estimated by S-transform [24], which is a combination of STFT and wavelet transforms (WT). The main advantages of S-transform are that it preserves the information on signal phase and provides a variable resolution that is similar to that of the WT. It is well known that the phase information is very important in the signal processing field [25,26,27,28,29,30,31]. In addition, the S-transform is a linear transform that can be used as an analysis tool or a synthesis tool, which is not the case with some of the bilinear transforms such as the Wigner–Ville distribution (WVD) [32]. Second, according to the local stationary length measured by the instantaneous frequency gradient (IFG), the proposed method gives different window widths at different time instants. Accordingly, a relationship between the window width and the IFG is addressed: a wide window is employed when the IF varies smoothly (IFG is small), and a narrow window is employed when the IF varies sharply (IFG is large). Furthermore, to increase robustness to the IF estimation error, the principal component analysis (PCA) [33,34,35] is used to estimate the IFG. It can be easily seen that the proposed method avoids one-dimensional search over the STFT window widths, and greatly reduces the amount of calculation. Third, the proposed method exploits the estimation strategy based on the O’Shea refinement procedure [36] to improve estimation accuracy. In this paper, numerous simulations are given to prove that the proposed method outperforms other estimation methods in terms of accuracy and calculation complexity.

This paper is organized as follows. The theoretical background of the QML is given in Section 2. In Section 3, the PPS-ASTFT principle is introduced. The computational cost and the performance of PPS-ASTFT are presented in Section 4. In Section 5, a brief conclusion is given. Regarding the notations used in this paper, $\arg\max\left(\cdot \right)$ denotes the argument of maximization operator; angle $\left(\cdot \right)$ denotes the phase of a complex number; unwrap $\left(\cdot \right)$ denotes unwrap operator; $\left|\cdot \right|$ denotes the modulus of the internal entity; $\left\lfloor \cdot \right\rfloor$ denotes the floor operator; ${\left(\cdot \right)}^{T}$ and ${\left(\cdot \right)}^{-1}$ represent the transpose and inverse, respectively.

## 2. QML Estimator

In this section, a brief introduction to QML and concepts used in our method are given. Suppose H is a set of window widths.

Firstly, we estimate the IF of the signal by detecting the ridge of STFT, which is defined by:

(2) $${\hat{\omega }}_{h}\left(n\right)=\arg\max_{\omega }\;\left|\mathrm{STF}T_{h}\left(n,\omega \right)\right|,$$

where h∈H and ${\hat{\omega }}_{h}\left(n\right)$ denotes the IF when the window width is *h*.

Secondly, we perform the polynomial regression [18,22] on the IF to coarsely estimate the coefficients $\left\{a_{k},k=1,\cdots ,M\right\}$ in the signal phase. The polynomial regression is defined by:

(3) $${\hat{a}}_{h}={\left(X_{}^{T}X_{}\right)}^{-1}X_{}^{T}\hat{\Omega },$$

where

(4) $${\hat{a}}_{h}={\left[{\hat{a}}_{1,h},{\hat{a}}_{2,h},\ldots ,{\hat{a}}_{M,h}\right]}^{T},$$

and

(5) $$\hat{\Omega }={\left[{\hat{\omega }}_{h}\left(1\right),{\hat{\omega }}_{h}\left(2\right),\ldots ,{\hat{\omega }}_{h}\left(N_{h}\right)\right]}^{T}.$$

$N_{h}$ is the number of effective signal length, and X is represented by:

(6) $$X=\left[\begin{array}{ccccc} 1 & t\left(1\right) & {\left[t\left(1\right)\right]}^{2} & \cdots & {\left[t\left(1\right)\right]}^{M-1}\\ 1 & t\left(2\right) & {\left[t\left(2\right)\right]}^{2} & \cdots & {\left[t\left(2\right)\right]}^{M-1}\\ \vdots & \vdots & \vdots & \ddots & \vdots \\ 1 & t\left(N_{h}\right) & {\left[t\left(N_{h}\right)\right]}^{2} & \cdots & {\left[t\left(N_{h}\right)\right]}^{M-1} \end{array}\right].$$

Thirdly, the parameter refinement procedure based on the O’Shea refinement strategy [36] is employed to improve the accuracy of the obtained coarse results, and the final estimation of the phase parameters, ${\hat{a}}_{h}^{final}={\left[a_{1,h}^{final},a_{2,h}^{final},\ldots ,a_{M,h}^{final}\right]}^{T}$, is obtained.

Fourthly, we choose another window width from H and repeat the above procedure until all widths are used. Then, from all estimations obtained with different window widths, we select the best one as an output result. However, the QML needs to perform a one-dimensional search over the set of window widths to obtain the optimal result and the computation cost high.

## 3. Proposed Estimator

In this paper, a low complexity method based on the ASTFT is proposed. The proposed method avoids both one-dimensional and multi-dimensional searches, and the error propagation problems that widely exist in the PPSs field. The proposed method mainly consists of five parts: firstly, IF of PPS is coarsely estimated by S-transform; secondly, IFG is estimated by the PCA; thirdly, window width of each time instant is estimated according to the relationship between IFG and window width; fourthly, the phase parameters of PPS are coarsely estimated by adaptive STFT and polynomial regression; fifthly, O’Shea refinement strategy is used for refining on the obtained coarse results.

### 3.1. Instantaneous Frequency Estimation by S-Transform

It is well known that the STFT uses a fixed sliding window to determine the tiling of time-frequency plane. Since the window is invariable, it suffers from a poor time-frequency resolution. An improved WT method uses an adaptive window whose width changes with frequency, thus a progressive resolution is provided. However, the time-scale plots produced by the WT are unsuitable for intuitive visual analysis and they suffer from a large mathematical burden. The S-transform is a combination of STFT and WT. The advantages of S-transform are that it can preserve the information on signal phase and provide a variable resolution similar to that of the WT. Moreover, by employing a scalable and variable Gaussian window and using the Fourier kernel, the S-transform provides the phase information referenced the time origin. The standard continuous S-transform of a signal $x\left(t\right)$ is defined by:

(7) $$\mathrm{ST}\left(t,f\right)=\int_{-\infty }^{\infty }x\left(\tau \right)w\left(\tau -t,f\right)\exp\left(-j2\pi f\tau \right)d\tau ,$$

where *t* denotes the time, *f* is the frequency, $w\left(\tau -t,f\right)$ is a scalable Gaussian window, which is represented by:

(8) $$w\left(\tau -t,f\right)=\frac{1}{\sigma \left(f\right)\sqrt{2\pi }}\exp\left(\frac{-{\left(\tau -t\right)}^{2}}{2\sigma^{2}\left(f\right)}\right),$$

and the standard deviation $\sigma \left(f\right)$ is a function of frequency *f*, which is defined by:

(9) $$\sigma \left(f\right)=\frac{1}{f}.$$

In previous equations, it can be easily seen that window width in the time domain is inversely proportional to the frequency, which further means that, in the time domain, the S-transform window is wider for lower frequency and narrower for higher frequency.

Then, the IF can be estimated by detecting the ridge of ST, which is represented by:

(10) $$f_{\mathrm{inst}\_\mathrm{ST}}\left(t\right)=\arg\max_{f}\;\left|\mathrm{ST}\left(t,f\right)\right|,$$

where $f_{\mathrm{inst}\_\mathrm{ST}}\left(t\right)$ denotes the IF.

The main purpose of our method is not to obtain the exact IF, namely, a small IF estimation error is considered as tolerable. During estimation of the IF, similarly to the Fourier transform, the aliasing issue influences estimation performance. In order to enhance algorithm adaptability, we can use the resolving aliasing method presented in [19].

### 3.2. Instantaneous Frequency Gradient Estimation

According to the above analysis, we know that a wide window should be employed when IF of the signal varies smoothly, and a narrow window should be employed when the IF varies sharply. Accordingly, the window width depends on the IFG of the signal, which is defined by:

(11) $$\nabla f_{\mathrm{inst}\_\mathrm{ST}}\left(t\right)=\frac{df_{\mathrm{inst}\_\mathrm{ST}}\left(t\right)}{dt}={f^{\prime }}_{\mathrm{inst}\_\mathrm{ST}}\left(t\right),$$

where ${f^{\prime }}_{\mathrm{inst}\_\mathrm{ST}}\left(t\right)$ denotes the IFG. In [37], the difference operator is presented for estimating the IFG. For a discrete signal with a sampling interval Δt, the discrete IFG at the *k*th time sampling point is represented by:

(12) $${f^{\prime }}_{\mathrm{inst}\_\mathrm{ST}}\left[k\right]=\frac{f_{\mathrm{inst}\_\mathrm{ST}}\left[k+1\right]-f_{\mathrm{inst}\_\mathrm{ST}}\left[k\right]}{\Delta t},$$

where $f_{\mathrm{inst}\_\mathrm{ST}}\left[k\right]$ relates to the *k*th IF measurement. It can be easily seen that the difference operator in Equation (12) is sensitive to the IF estimation error, especially in noisy environments because only two IF measuremets are used to evaluate the IFG. According to [33,34,35], the PCA is a standard tool in multivariate data analysis to reduce the number of dimensions, while retaining as much as possible data variation. For a time-frequency signal, there are two features including time and IF. By performing the PCA operation on the time-frequency signal, we can obtain the principal components, and the slope of the first principal component vector approximates to the IFG [38]. For example, to get the ${f^{\prime }}_{\mathrm{inst}\_\mathrm{ST}}\left[k\right]$ of *k*th point, we utilize $\left(2K+1\right)$ estimated IFs, which are obtained by Section 3.1, to form a two-dimensional matrix $\left[T,F\right]$, where $T={\left[\left(k-K\right)\Delta_{t},\left(k-K+1\right)\Delta_{t},\cdots ,\left(k+K\right)\Delta_{t}\right]}^{T}$ and $F={\left[f_{\mathrm{inst}\_\mathrm{ST}}\left(k-K\right),f_{\mathrm{inst}\_\mathrm{ST}}\left(k-K+1\right),\cdots ,f_{\mathrm{inst}\_\mathrm{ST}}\left(k+K\right)\right]}^{T}$. Then, the covariance matrix of the two-dimensional measurements is given by:

(13) $$C=\left[\begin{array}{cc} C_{\mathrm{TT}} & C_{\mathrm{TF}}\\ C_{\mathrm{FT}} & C_{\mathrm{FF}} \end{array}\right]≜\left[\begin{array}{cc} \mathrm{cov}\left(T,T\right) & \mathrm{cov}\left(T,F\right)\\ \mathrm{cov}\left(F,T\right) & \mathrm{cov}\left(F,F\right) \end{array}\right].$$

When we perform the eigenvalue decomposition operator on C, the eigenvalue and eigenvector are obtained as:

(14) $$e=\left[\begin{array}{cc} e_{11} & e_{12}\\ e_{21} & e_{22} \end{array}\right]\;\mathrm{and}\;\lambda ={\left[\lambda_{1},\lambda_{2}\right]}^{T}.$$

Suppose $\lambda_{1}$ is the larger eigenvalue, then the relevant eigenvector $e_{1}={\left[e_{11},e_{12}\right]}^{T}$ is the principal component vector. Thus, the IFG ${f^{\prime }}_{\mathrm{inst}\_\mathrm{ST}}\left[k\right]$ is approximated by the slope of $e_{1}$, which is defined by:

(15) $${f^{\prime }}_{\mathrm{inst}\_\mathrm{ST}}\left[k\right]\approx \frac{e_{12}}{e_{11}}.$$

The difference operator in Equation (12) can be viewed as a special case of the PCA with using only two IF measurements to calculate the IFG. Compared with the difference operator, the explained strategy can obtain better anti-noise performance by using more IF measurements (large *K*). It is noteworthy that, for different PPSs, the optimal *K* values are also different. The optimal *K* value is large for low order and slowly changing IF of PPS, and small for high order and quickly changing IF of PPS. If there is no prior knowledge about the PPS, *K* is set to 2 based on the experience. In that case, the anti-noise performance loss is about 2 dB compared to that for an optimal *K* value. However, the performance of the proposed method still outperforms most of the PPS algorithms. Finally, in order to improve the anti-noise ability of the proposed method, the classical polynomial regression [18,22] is applied to IFG.

### 3.3. Adaptive Window Width Estimation

Suppose $\Delta_{t}$ and $\Delta_{\omega }$ are the time resolution and frequency resolution, respectively. According to the Heisenberg uncertainty principle [39], we have:

(16) $$\Delta_{t}\Delta_{\omega }\geq \frac{1}{2}.$$

When the value of $\Delta_{t}\Delta_{\omega }$ is equal to 1/2, it is called the Heisenberg box. Since the Heisenberg box is constant, time and frequency resolution cannot be maximized at the same time. Fortunately, a reasonable harmonization between time and frequency resolution by optimizing the window width using the signal characteristics is presented in [40].

In [40], for purely frequency modulated signals, the optimal window width relates to the IFG. If the purely frequency modulated signal is defined by:

(17) $$y\left(t\right)=\exp\left(j\varphi \left(t\right)\right),$$

where $\varphi \left(t\right)$ denotes phase function. Then, the theoretical optimal window width $T_{t}$ can be calculated by:

(18) $$T_{t}\approx \frac{1}{\sqrt{2\left|\varphi^{\prime }\left(t\right)\right|}}=\frac{1}{\sqrt{4\pi \left|{f^{\prime }}_{\mathrm{inst}\_\mathrm{ST}}\left(t\right)\right|}},$$

where ${f^{\prime }}_{\mathrm{inst}\_\mathrm{ST}}\left(t\right)$ is the IFG.

In the proposed method, the STFT is defined by:

(19) $$\mathrm{STFT}\left(t,f\right)=\int_{-\infty }^{\infty }x\left(\tau \right)\omega_{\sigma }\left(\tau -t\right)\exp\left(-j2\pi f\tau \right)d\tau ,$$

where $\omega_{\sigma }\left(t\right)$ is the Gaussian window kernel function, which is defined by:

(20) $$\omega_{\sigma }\left(t\right)=\frac{1}{\sqrt{2\pi }\sigma }\exp\left(-\frac{t^{2}}{2\sigma^{2}}\right),$$

where σ is the dilation parameter.

According to [41], the Gaussian window width is defined as its full width at a half maximum value (FWHM) value, which is represented by:

(21) $$T_{G}=2\sigma \sqrt{2\ln2}.$$

Thus, $T_{G}=2T_{t}$. By substituting Equation (21) into $T_{G}=2T_{t}$, we get:

(22) $$\sigma =\frac{T_{t}}{\sqrt{2\ln2}}.$$

As a result, the actual adaptive Gaussian window width can be obtained by σ.

### 3.4. Coarse Estimator

We perform the ASTFT operator on the PPS by using an adaptive window width. The IF can be estimated by:

(23) $$f_{\mathrm{inst}\_\mathrm{ASTFT}}\left(t\right)=\arg\max_{f}\;\left|\mathrm{ASTFT}\left(t,f\right)\right|.$$

Although this estimator is biased, the IF contains phase coefficients $\left\{a_{k},k=1,\cdots ,M\right\}$ information. When the classical polynomial regression [18,22] is applied to $f_{\mathrm{inst}\_\mathrm{ASTFT}}\left(t\right)$, the coarsely estimated coefficients $\left\{{\hat{a}}_{k},k=1,\cdots ,M\right\}$ can be obtained. The polynomial regression is defined by Equations (3)–(6). It is noticed that $\hat{\Omega }$ denotes angular frequency in Equation (3).

According to [18,22], the polynomial regression is a biased estimator, thus it is very difficult to derive a closed form expression for estimator variance. Due to the bias, the obtained estimation can be treated as a coarse result. Thus, we should use a fine estimation strategy to improve estimation accuracy.

### 3.5. Refinement Strategy

In this section, we use the O’Shea refinement strategy proposed in [36] to refine the obtained coarse results. This O’Shea refinement is divided into three steps.

Firstly, the PPS is dechirped by the coarse results obtained in Section 3.4, and then the low-pass filtering operation is performed on the residual signal. The processing is represented by:

(24) $$\hat{x}\left(t_{l}\right)=\frac{1}{L}\sum_{p=-lL+\left(L-1\right)/2}^{lL-\left(L-1\right)/2}x\left(p\right)\exp\left(-j\sum_{k=1}^{M}{\hat{a}}_{k}{\left(p\Delta t\right)}^{k}/k\right),$$

where *L* denotes the filter length, $Q=\left\lfloor N/L\right\rfloor$, $\left\lfloor \cdot \right\rfloor$ is the floor operator, and $t_{l}=\left(l+Q/2\right)L\Delta t-N\Delta t/2$, $l\in \left[-Q/2,Q/2\right]$. The filtering in this step is used to increase the SNR explained in [36].

Secondly, the unwrapping operation is performed on the phase of $\hat{x}\left(t_{l}\right)$, thus:

(25) $$V=\mathrm{unwrap}\left(\mathrm{angle}\left(\hat{x}\left(t_{l}\right)\right)\right).$$

Then, V is modeled as a new PPS in the noise with phase parameters $a_{v}={\left[a_{0}\;\delta a_{1}\cdots \delta a_{M}\right]}^{T}$, where $\delta a_{k}=a_{k}-{\hat{a}}_{k}$, k=1,⋯,M. Then, these new phase parameters are estimated according to:

(26) $${\hat{a}}_{v}={\left[{\hat{a}}_{0}\;\hat{\delta }a_{1}\cdots \hat{\delta }a_{M}\right]}^{T}={\left(G^{T}G\right)}^{-1}G^{T}V,$$

where G is the rectangular Vandermonde matrix, which is represented as:

(27) $$G=\left[\begin{array}{ccccc} 1 & t_{-Q/2} & {\left[t_{-Q/2}\right]}^{2} & \cdots & {\left[t_{-Q/2}\right]}^{M}\\ 1 & t_{-Q/2+1} & {\left[t_{-Q/2+1}\right]}^{2} & \cdots & {\left[t_{-Q/2+1}\right]}^{M}\\ \vdots & \vdots & \vdots & \ddots & \vdots \\ 1 & t_{Q/2} & {\left[t_{Q/2}\right]}^{2} & \cdots & {\left[t_{Q/2}\right]}^{M} \end{array}\right].$$

Thirdly, the final estimates of phase parameters are obtained using the following relations:

(28) $${\hat{a}}_{k}^{final}={\hat{a}}_{k}^{}+k\frac{\hat{\delta }a_{k}^{}}{L^{k}},\;k=1,2,\cdots M,$$

(29) $${\hat{a}}_{0}^{final}={\hat{a}}_{0}.$$

The PPS-ASTFT method can be described by the steps given in Algorithm 1.

 **Algorithm 1** **Input:** The PPS signal is $x\left(t\right)$.Step 1:Estimate PPS IF $f_{\mathrm{inst}\_\mathrm{ST}}$ by detecting the range of S-transform.Step 2:Perform Equations (13) and (14) on $f_{\mathrm{inst}\_\mathrm{ST}}$ to obtain the eigenvector e. Then, the IFG ${f^{\prime }}_{\mathrm{inst}\_\mathrm{ST}}$ is estimated by Equation (15).Step 3:The theoretical optimal window width $T_{t}$ is determined by Equation (18). Using the relationship given in Equation (22), $\sigma =\frac{T_{t}}{\sqrt{2\ln2}}$, the actual Gaussian window width for each time instant is estimated.Step 4:Based on the adaptive Gaussian window width for each time instant, instantaneous frequency $f_{\mathrm{inst}\_\mathrm{ASTFT}}$ can be estimated by Equation (23). Then, the coarsely estimated results $\left\{{\hat{a}}_{k},k=1,\cdots ,M\right\}$ are obtained by Equation (3).Step 5:Estimate the cofficients $\left\{{\hat{a}}_{k}^{final},k=0,\cdots ,M\right\}$ by using the refinement strategy defined by Equations (24)–(29). **Output:** The estimation coefficients $\left\{{\hat{a}}_{k}^{final},k=0,\cdots ,M\right\}$.

In addition, when the order of PPS is unknown, we can use the strategy presented in [20]. For this case, we should search over the set of potential signal orders. The algorithm can be divided into three steps. Firstly, suppose M is the set of potential signal orders, we perform Step 1 to Step 5 in Algorithm 1 on the PPS for each order m∈M, and we can obtain the corresponding estimation coefficients $\left\{{\hat{a}}_{k,m}^{final},1\leq k\leq m\right\}$. Secondly, evaluate the quasi maximum likelihood function presented in [18,19,20], which is defined by:

(30) $$J\left(m\right)=\left|\sum_{n}x\left(n\right)\exp\left(-j\sum_{k=1}^{m}{\hat{a}}_{k,m}^{final}{\left(n\Delta t\right)}^{k}/k\right)\right|,$$

where ${\hat{a}}_{k,m}^{final}$ denotes the *k*th $\left(1\leq k\leq m\right)$ order coefficient. Thirdly, choose the value of signal order, which maximizes the quasi maximum likelihood function, as the final estimation of the PPS order, and the corresponding coefficients are the final estimation of the PPS coefficients.

## 4. Numerical Simulations

In this study, we consider a signal of the following form:

(31) $$x\left(t\right)=A\exp\left(j\sum_{k=1}^{M}a_{k}t^{k}/k+ja_{0}\right)+v\left(t\right),\;t\in \left[-T/2,T/2\right),$$

where $v\left(t\right)$ denotes the additive Gaussian noise with $\mathrm{SNR}\in \left[-5,25\right]$ dB, *T* is the signal duration, and *M* = 5, 6 and 7. In order to prove the effectiveness of the proposed algorithm, the parameters selection strategy is similar to that in [19]. The amplitude and initial phase are A=1, $a_{0}=20$, respectively. The length of signals is N=256, and sampling interval is Δt=1/256. The phase parameters are given in Table 1.

According to the analysis in [13,17,18], the PHAF and the PHAF-CPF show much better identifiability and noise rejection capability than the other PD-based methods. Moreover, the QML outperforms other TFR-based methods. Therefore, we chose these three methods as references.

### 4.1. Simulation Time

The main implementation procedures of the proposed method include the ST operator, the PCA operator, the STFT operator and the O’Shea refinement strategy. Assume the search space is *N*. Due to the computational efficiency of the Fast Fourier transform (FFT) used to calculate the ST, the total number of operations is $O\left(N+N\log_{2}N\right)$ [42]. The PCA function requires $O\left(N\left(\left(2K+1\right)F^{2}+F^{3}\right)\right)$, where *F* is the number of features. In this paper, the features contain only time and frequency, thus *F* is equal to two. Since the matrices ${\left(X_{}^{T}X_{}\right)}^{-1}X_{}^{T}\hat{\Omega }$ in Equation (3) and ${\left(G^{T}G\right)}^{-1}G^{T}V$ in Equation (26) can be calculated in advance and stored in memory, the most complicated procedure in parameter estimation is the STFT evaluation with $O\left(N^{2}\log_{2}N\right)$ operations. Thus, the total cost of the proposed method is $O\left(N^{2}\log_{2}N\right)$.

Table 2 lists the average simulation time of PHAF, PHAF-CPF, QML, and PPS-ASTFT. In this paper, the number of lag sets of the PHAF and PHAF-CPF is set to 3. According to [43], the number of QML windows is set to N/8. The simulations are completed on a computer with an Intel Core G2030 (3.00 GHz) and 12 GB memory.

In Table 2, it can be seen that the CPU time of PPS-ASTFT is significantly less than that of QML, and close to that of PHAF-CPF. Among these methods, the PHAF is the fastest one. It is noteworthy that simulation time of both PHAF and PCPF-HAF increases with the signal order. According to the analysis in [13,17], as the signal order increases, more PD operations are used, and those lead to increasing of calculation.

### 4.2. Performance Analysis

According to the metrics used for other estimation algorithms [18,19,20,22,36], we utilize the mean-squared error (MSE) defined as follow to evaluate accuracy of the proposed algorithm:

(32) $$\mathrm{MS}E_{k}=\frac{1}{N_{\mathrm{trials}}}\sum_{r=1}^{N_{\mathrm{trials}}}{\left({\hat{a}}_{k,r}-a_{k}\right)}^{2},\;k\in \left[0,\;M\right],$$

where $N_{\mathrm{trials}}$ denotes the number of trials, and here $N_{\mathrm{trials}}$ = 500, and ${\hat{a}}_{k,r}$ is the estimated value of $a_{k}$. According to the analysis in [18], the nonlinear techniques for higher order PPSs produce results above the CRLB for a couple of dBs. Therefore, in order to prove effectiveness of the proposed method, the same refinement strategy was used for both PHAF and PCPF-HAF to improve their accuracies.

**Figure 1:**
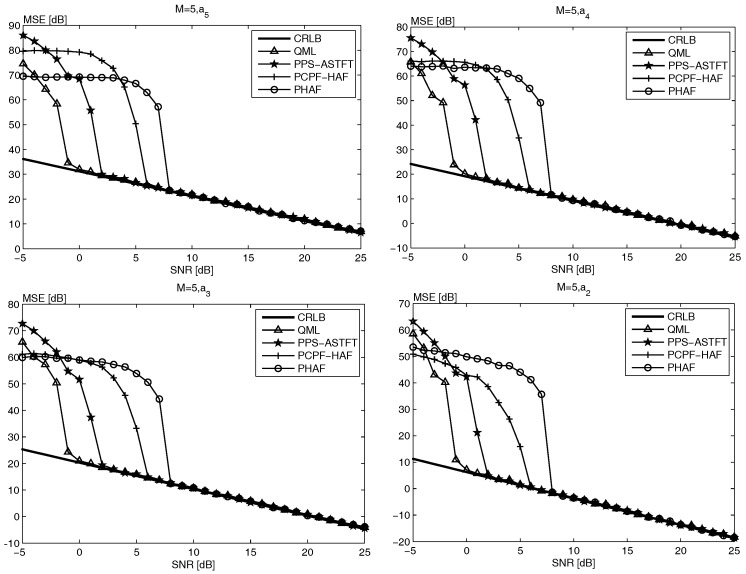
The MSE of the four highest order coefficients of the 5th-order PPS.

In Figure 1, the MSEs of the four highest order characteristic parameters of 5th order PPS are shown, wherein it can be seen that the TFR-based estimators, QML and PPS-ASTFT, outperform the PD-based estimators, PHAF and PCPF-HAF. Based on the analyses in [13,17], the PD-based estimator suffers from error propagation and PD operation, which lead to a high SNR threshold. The SNR thresholds of PHAF and PCPF-HAF are 8 dB and 6 dB for M=5, respectively. Compared to the PHAF, the PCPF-HAF performs less PD operation, thus its SNR threshold is 2 dB lower. Although the simulation time of PHAF and PCPF-HAF of 5th order PPS is less than that of proposed method, the SNR thresholds are much higher. In the TFR-based estimator, all parameters are estimated simultaneously, therefore, there is no error propagation problem. Moreover, as explained in [36], the filtering tends to cause the SNR to increase, and the O’Shea refinement strategy tends to improve estimation accuracy. Hence, the QML and PPS-ASTFT can obtain better estimation performance than the PHAF and PCPF-HAF methods. In Figure 1, the SNR thresholds of QML and PPS-ASTFT are 0 dB and 2 dB, respectively, and they are much lower than those of PHAF and PCPF-HAF. Although the SNR threshold of the proposed method is slightly higher than that of the QML method, the simulation time of the proposed method given in Table 1 is much less because the QML method needs to repeat the searching and estimating operation, as described in Section 2.

**Figure 2:**
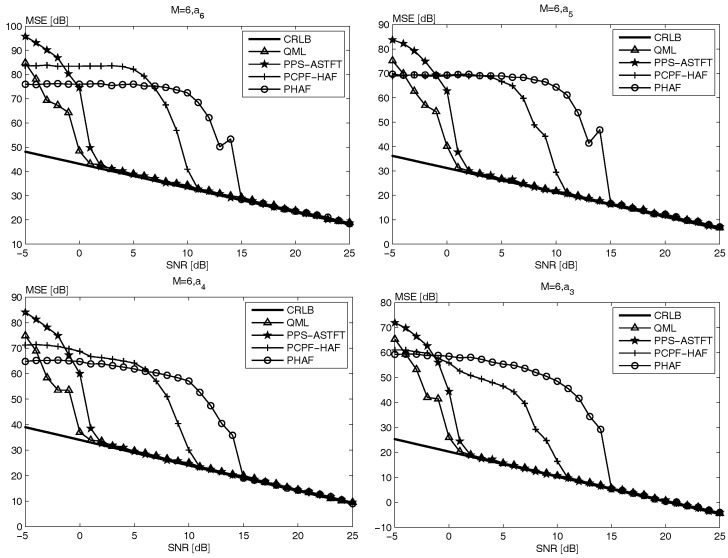
The MSE of the four highest order coefficients of the 6th-order PPS.

**Figure 3:**
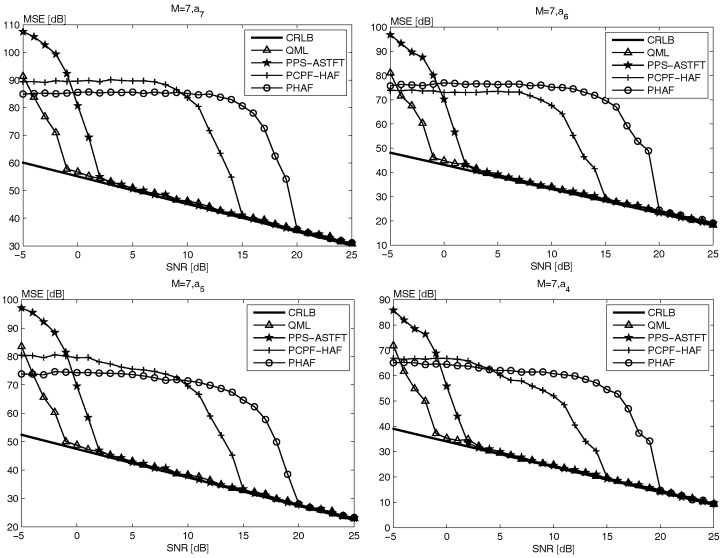
The MSE of the four highest order coefficients of the 7th-order PPS.

Figure 2 and Figure 3 show the same conclusions. In addition, it is noteworthy that the SNR threshold of both PHAF and PCPF-HAF becomes higher with the increase of signal order. According to the analysis in [13,17,19], with the signal order increasing, the error propagation and PD operation affect the performance more seriously. Moreover, since simulation time of both PHAF and PCPF-HAF increases with the signal order, as seen in Table 1, therefore the performance of PHAF and PCPF-HAF is worse when the PPS order is higher. However, it is easily seen form Table 1 and Figure 1, Figure 2 and Figure 3, the performance of both PPS-ASTFT and QML is almost unaffected by the signal order. According to the analysis on computational cost and estimation performance, the proposed method is more suitable for practical applications than other methods.

## 5. Conclusions

In this paper, a low complexity method named PPS-ASTFT is proposed to estimate the PPS parameters. In the proposed algorithm, the IF is estimated by S-transform, which can preserve information on signal phase and provide a variable resolution. The width of the ASTFT analysis window is calculated by the PCA, which is robust to the noise. In the parameters estimation, by using the refinement strategy, the estimation accuracy is improved. Through the simulations, it is shown that the proposed method costs a little more time than PCPF-HAF, but the SNR threshold of proposed method is significantly lower than that of PCPF-HAF. Although the PHAF costs less time than the proposed method, the performance of PHAF is worse. In addition, the SNR threshold of the proposed method is slightly higher than that of the QML method, but the simulation time is much less than that of QML because a one-dimensional search over the STFT window widths is avoided. Therefore, through comprehensive analyses, the PPS-ASTFT is more suitable for practical applications than other methods. In the future, our work will be mainly focused on the follow issues: (i) since the S-transform suffers from poor energy concentration in some situations, while recent development in this field has enhanced the energy concentration, but the computational cost is still unsatisfactory, thus we will focus on enhancing the energy concentration in the time–frequency domain with a low complexity; (ii) since the value of K of the PCA affects the estimation performance, we will study the relationship between that value and estimation performance; and, (iii) although the proposed method is an adaptive method, the adaptability is not great. To improve the adaptability, we will study the adaptive method, such as the method presented in [29].

## Figures and Tables

**Table 1:** The phase coefficients of signals in numerical example.

*M*	$a_{7}$	$a_{6}$	$a_{5}$	$a_{4}$	$a_{3}$	$a_{2}$	$a_{1}$	$a_{0}$
M=5			246.096	0.0302	–737.4794	39.995	722.1379	20
M=6		0.3007	165.2	–0.0867	–626.8528	5.006	678.5557	20
M=7	–26.7439	–0.0036	122.0581	0.001	–248.0279	50	550.7961	20

**Table 2:** Simulation time.

Estimation Algorithm	M = 7	M = 6	M = 5
PHAF	0.0516	0.0423	0.0362
PHAF-CPF	0.2032	0.1851	0.1702
QML	1.5183	1.5101	1.4504
PPS-ASTFT	0.2121	0.1991	0.1921
